# Towards an FDA-cleared basophil activation test

**DOI:** 10.3389/falgy.2022.1009437

**Published:** 2023-01-09

**Authors:** Oral Alpan, Richard L. Wasserman, Theodore Kim, Amy Darter, Atul Shah, Douglas Jones, Donald McNeil, Henry Li, Laura Ispas, Melinda Rathkopf, Elena Perez, Dareen Siri, Maeve O’Connor, Matthew Plassmeyer, Kimberly Romito, Christina Pettibone, Sean O’Reilly, Søren Ulrik Sønder, Gerald Marti

**Affiliations:** ^1^ Amerimmune, Fairfax, VA, United States; ^2^ Medical City Children’s Hospital, Dallas, TX, United States; ^3^ Allergy Partners of Northern Virginia, Chantilly, VA, United States; ^4^ Oklahoma Institute of Allergy, Asthma and Immunology, Oklahoma City, OK, United States; ^5^ New York Food Allergy and Wellness Center, Centereach, NY, United States; ^6^ Tanner Clinic, Layton, UT, United States; ^7^ OptiMed, Columbus, OH, United States; ^8^ Institute for Asthma and Allergy, Wheaton, MD, United States; ^9^ Allergy, Asthma and Immunology Institute, Leesburg, VA, United States; ^10^ Allergy Asthma and Immunology Center of Alaska, Anchorage, AK, United States; ^11^ Allergy Associates of the Palm Beaches, Palm Beach, FL, United States; ^12^ Midwest Allergy Sinus Asthma, Normal, IL, United States; ^13^ Allergy, Asthma and Immunology Relief of Charlotte, Charlotte, NC, United States; ^14^ New Columbia Capital, LLC, Arlington, VA, United States

**Keywords:** basophil activation test, food and drug administration, peanut allergy, food allergy, laboratory developed test

## Abstract

Food allergy is a global health problem affecting up to 10% of the world population. Accurate diagnosis of food allergies, however, is still a major challenge in medical offices and for patients seeking alternative avenues of diagnosis. A flawless test to confirm or rule out a food allergy does not exist. The lack of optimum testing methods to establish precise clinical correlations remains a major obstacle to effective treatment. Certain IgE measurement methods, including component testing, have received FDA clearance, but they have been used primarily as an analytical tool and not to establish clinical correlations. Most allergy tests are still carried out within the laboratory, and skin tests outside a laboratory setting that are used for food allergy diagnosis rely on non-standardized allergens, according to the FDA definition. Epitope mapping and basophil activation test (BAT) have recently been proposed as a means of establishing better clinical correlations. Yet neither have received FDA clearance for widespread distribution. Of the two methods, the BAT has the advantage of being a functional assay. Over the past few years, several large private practice groups in the United States, have developed BAT as a clinical assay and have started using it in patient care. Given this clinical experience, the vast number of papers published on BAT (more than 1,400 as of 2022) and the trend toward increasing FDA regulation, it is essential to understand the roadmap for regulatory clearance of this assay.

## 1. Introduction

The prevalence of food allergies in the United States is between 4% and 10%. Milk, tree nuts, peanut, egg, shellfish, fish, soy, and wheat make up approximately 95% of the total (1, 2). A major challenge in identifying food allergies stems from the lack of readily available and accurate in-vitro clinical laboratory tests (IVCT) that correlates with patients’ clinical presentations (3, 4). The first steps in the work-up of food allergies is establishing a good clinical history and conducting skin testing and allergen specific IgE measurements (5). Although these testing procedures have demonstrated good sensitivity for detecting allergic individuals, their specificity is low, and they lack reliable threshold values (6, 7). For this reason, physicians treating food allergies often base their decisions on personal experience, which can vary significantly, and on anecdotal information.

**Figure 1:**
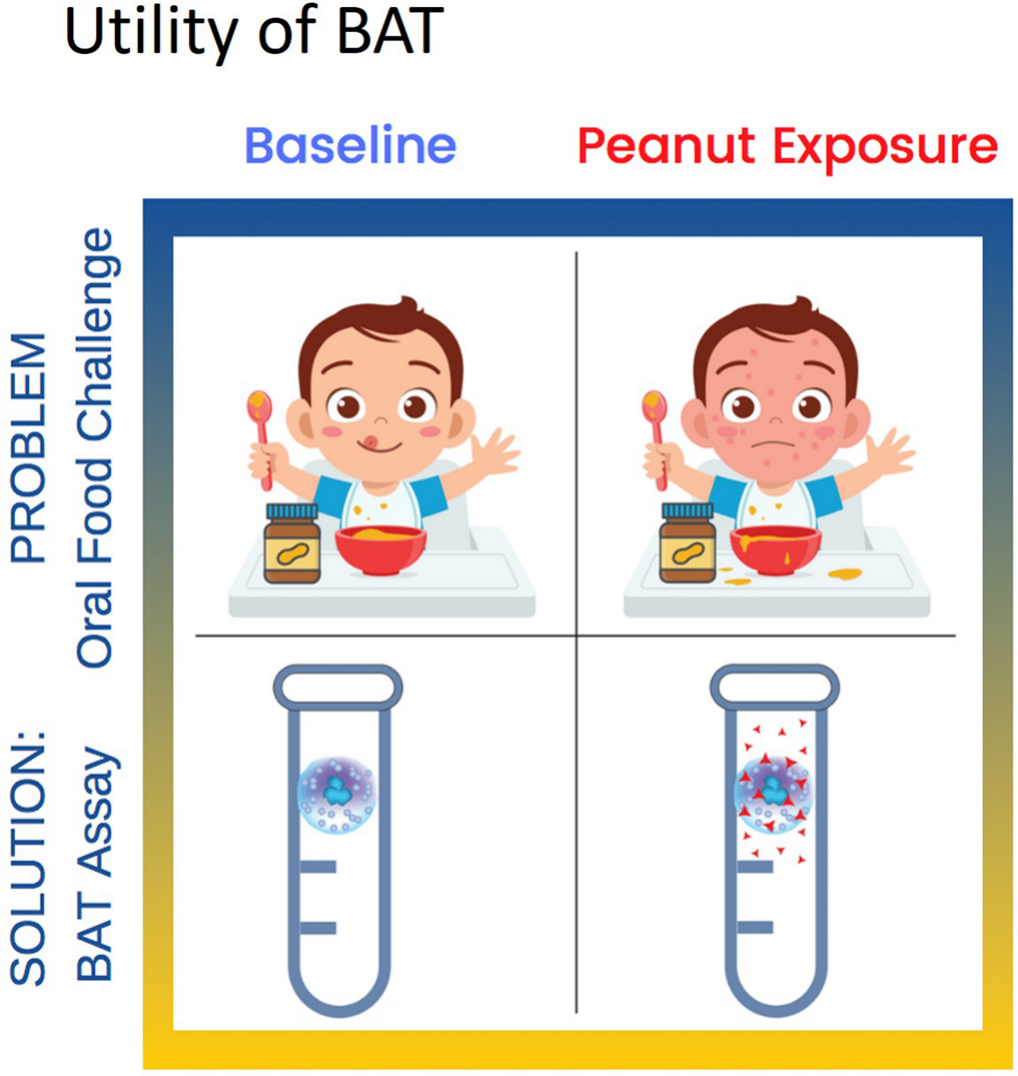
Basophil activation test is a good measure to assess clinical reactivity to prevent unnecessary food challenges.

At the present time, an oral food challenge (OFC) is the gold-standard for confirming a food allergy. This method of confirming food allergy diagnosis, however, has serious disadvantages. It can be labor intensive, costly and carries the risk of allergic reaction in an office setting (8). Furthermore, the test can be a source of anxiety for patients and their families because of the risk of such a reaction. Therefore, a new test is needed, especially for the most common and important food allergies (Figure 1).

Peanut allergy is an important health problem because it is among the most common food allergies. Depending on the geographic location, peanut allergy has a prevalence of 0.1%–1.5%. Currently, peanut allergy is the only one for which an FDA approved drug is available (9–12). The basophil activation test (BAT) is well suited for detecting peanut allergy. BAT can very effectively distinguish various clinical phenotypes of peanut allergy (e.g., anaphylactic vs. non-anaphylactic reactions) (13–15). In the context of cross-reactivity syndromes (e.g., wheat and grass pollen), results of BAT can overestimate clinical allergy. For peanut allergen, this cross-reactivity is less of a problem for seed storage proteins, but not for lipid transfer proteins (LTP) in certain geographical areas (16).

A significant clinical challenge is differentiating between clinical food allergy and sensitization, the latter of which can be seen in up to 10% of the population (17). Given advances in managing food allergies, it has become more important to identify those with real clinical allergy versus sensitization (positive test but no clinical reaction) and to predict the type of allergic reaction. These issues are important in decision- making for treatments. Tests that can separate sensitivity from clinical allergy with clear cut-off values are in great need (18).

## 2. Basic methodological aspect of BAT

BAT is a flow cytometry assay which measures the expression of activation markers on the basophil surface and the basophil activation process through IgE cross-linking. The hallmark of BAT, detection of CD63 on the basophil cell surface, was first discovered by Edward Knol in 1991 (19). In his report, human basophils were activated with anti-IgE and chemotactic peptide, N-formyl-methionyl-leucyl-phenylalanine (fMLP). Both these methods of stimulation induced a distinct increase in expression of the CD63 on the surface of basophils. Cell surface CD63 was detected by the monoclonal antibody (MAb) 435. Time dependent kinetics of CD63 up-regulation as detected by Mab 435 binding to basophils correlated strongly with histamine release. This indicates degranulation. A comprehensive review of the historical, technical, and clinical aspects of BAT has recently been published (20).

**Figure 2:**
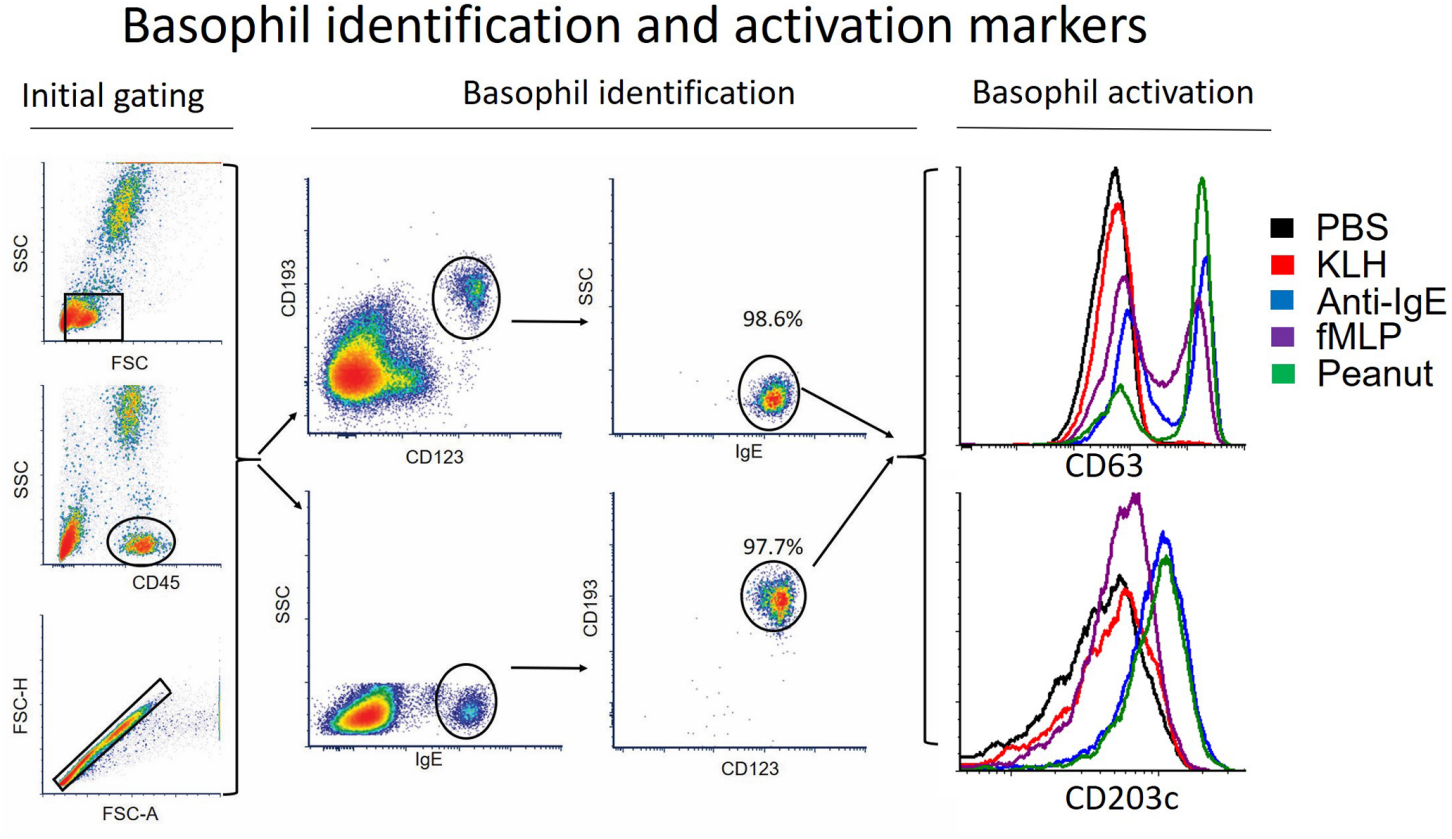
Determine basophil activation by flow cytometry. The initial gating aims at isolating singlets and removing eosinophiles, neutrophils and to some extent monocytes and DC's. The subsequent basophil identification where basophils are identified based on their surface markers. Here two approaches are shown, CD123/CD193 and IgE/SSC. Lastly the activation determined by CD63 and CD203c are shown for the two negative controls (PBS and KLH), the two positive controls (Anti-IgE and fMLP) and the peanut allergen.

The first step in performing a BAT is identification of basophils in whole blood. Two approaches to identify basophils in whole blood is shown in Figure 2. It is possible to combine the two approaches for increased stringency. Once basophils are identified, spontaneous activation and the effect of an inert antigen on basophils is tested. For spontaneous activation, basophil surface markers are stained in the absence of any allergens. To assess basophil response to an inert antigen one that humans are not sensitized or allergic to is needed. For this purpose, we have implemented the use of keyhole limpet hemocyanin (KLH). This antigen is a metalloprotein found in deep sea giant keyhole limpet, off the coast of California. There is very little cross-reaction with any other allergen and humans are rarely sensitized to it, making it a perfect negative control allergen (21, 22).

The second step is the use of a positive control that verifies the viability and responsiveness of the basophils. As positive controls, both fMLP and anti-IgE are used. Activation of basophils independently of the IgE-FcεRI pathway by fMLP is important to verify whether basophils in the blood sample are healthy to go through BAT (4, 23). These controls are critical in evaluating degraded/expired allergens, interference with basophil surface receptors or signaling, inhibition by various plasma proteins, and poor response due to baseline activation of the cells as well as non-responsive (anergic) basophils (4, 24).

The third step in BAT is to perform the allergen dose (concentration)-response curves. These dose-response curves can be interpreted with metrics such as basophil sensitivity, median effective concentration, area under the curve (AUC) and basophil reactivity (25). Each of these reporting methods have been validated with proper cut-off values based on the patient population tested and detailed summaries of these methods have been published (26, 27). Each clinical laboratory establishes optimal allergen-specific cutoff limits for the specific question the test is being used in the clinic. Clinical relevance of different types of dose-response curves (i.e., bell shape, linear, bimodal and plateau) still need further investigation.

Additional basophil activation markers have also been identified, including CD203c, diamine oxidase measurement of intracellular histamine, CD107a, CD13 and CD164, among others. However, CD63 has remained the most widely used market (28–30). In contrast to CD63, many of these other activation markers may be up-regulated in response to non-degranulation stimuli, hence limiting their clinical utility (31). It is possible that these additional markers may have value in defining clinical desensitization or basophil tolerance induction (32, 33).

### 2.1. The use of BAT in clinical practice and its interpretation

**Figure 3:**
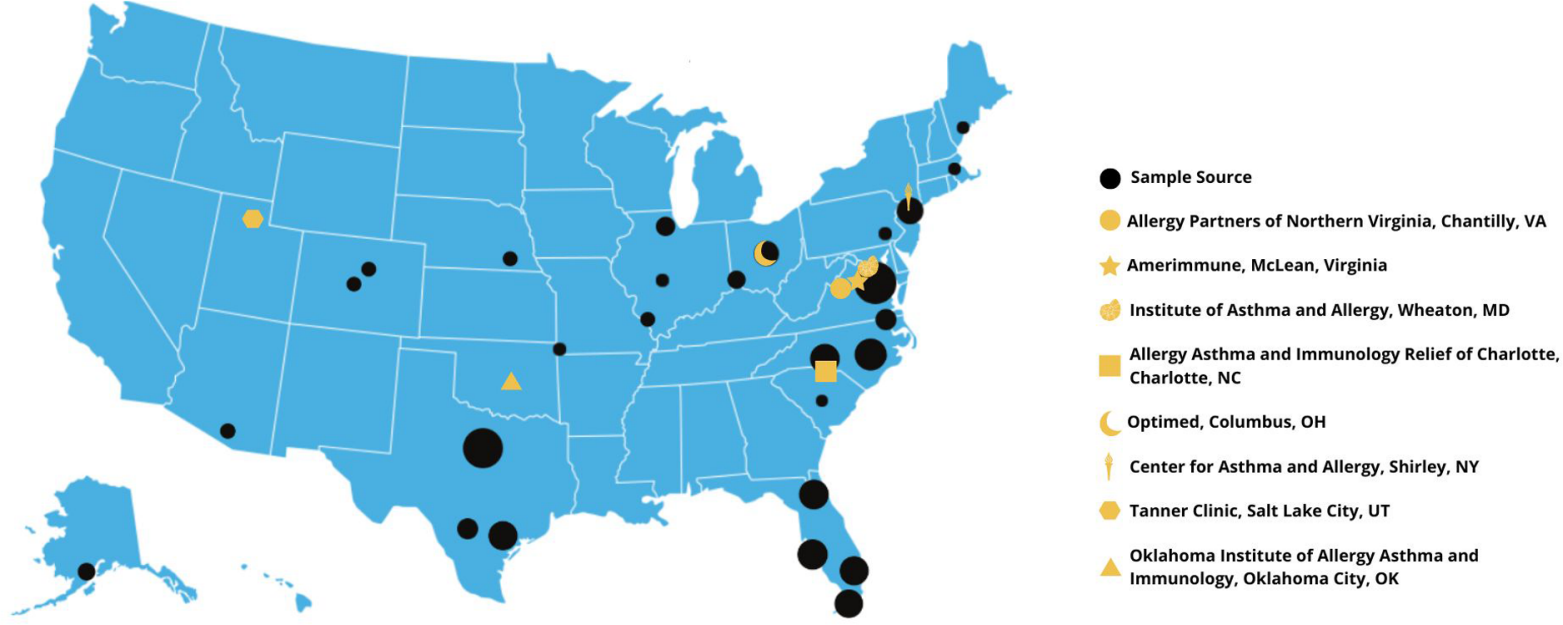
Sample sources and laboratory location for BAT in the United States. Dark circles represent sources for the blood samples. Size of each circle represent sample size from each location. Total samples are over 1,000 between year 2018 and 2021. The yellow symbols represent CLIA/CAP accredited laboratory locations capable of performing BAT as an LDT.

Although BAT is a well-established, robust, and reproducible assay with great potential for physicians in identifying allergies, its use in clinical practice has been limited by several factors. Differences in the infrastructure and expertise of the laboratories where tests are performed, the diversity of clinical reporting methods, differences in preparation and sources of the allergens, and a lack of clear clinical guidelines in how to use BAT in the diagnostic algorithms are some reasons for the limited use. Each clinical laboratory has developed its own methods and reporting protocols. Currently, there are eight laboratories in the United States that perform BAT as a CLIA (Clinical Laboratory Improvement Amendments) approved CAP (College of American Pathologists) accredited assay (Figure 3). All these laboratories are within private Allergy/Immunology practices. There is no country-wide standardization because the FDA allows regulations to be devised by the states. In other countries, the use of BAT is standardized because it is regulated by national-level authorities. BAT has been widely used as a research test and a clinical diagnostic tool in Sweden, Spain, Germany, Denmark, Italy, and South Africa under such a regulatory system (20).

BAT has the potential to be a more effective diagnostic tool in the U.S. if a consistent nationwide standard under the FDA could be established. To develop such a standard, it is important to determine at which point of the diagnostic process this test should be performed. The most practical utility of BAT is to guide a food challenge decision (20). BAT can also help determine appropriate candidates for oral food immunotherapy, natural resolution of food allergy and monitoring response to immunotherapy. Since BAT is a functional assay and uses multiple allergen concentrations to obtain dose response curves, the information obtained from this test allows for a much more detailed picture of the response to the food allergen.

In a recent and well documented study, basophil allergen threshold sensitivity (the lowest concentration of peanut allergen activating basophils) and IgE antibodies to peanut allergen were compared to double-blind placebo-controlled food challenge, (DBPCFC). Over 90% of children who failed DBPCFC also showed reactive BAT after stimulation with peanut or Ara h 2, indicating excellent sensitivity. Of those with a negative DBPCFC, approximately 70% were negative in basophil activation with peanut and Ara h 2. Three children with negative food challenges with positive serum peanut specific IgE and Ara h 2 demonstrated positive BAT to both allergens. All children with negative basophil activation passed DBPCFC to peanut indicating excellent specificity of the test (34, 35). Larger studies with similar clinical design are needed to further validate peanut—BAT before regulatory clearance can be obtained (34). Such studies will most likely require several hundred subjects to obtain robust sensitivity and specificity data.

In certain cases, BAT can predict the severity of clinical reactions as well as the prognosis of the patient's food allergy (36). In cases of oral food immunotherapy, BAT can predict thresholds of reactivity to help determine dosing of the patients as well as degrees of tolerance. The reporting of these recommendations, however, will require controlled clinical trials and the establishment of a federal standard that is country wide.

Although BAT for peanut has been studied the most, there is also data on foods such as cow's milk, egg, wheat, tree nuts, shellfish, apple carrot and celery among others. For example, the current diagnostic tests for cow's milk allergy include sIgE (sensitivity 87%, specificity 48%) and SPT (sensitivity 88%, specificity 68%). BAT has a higher sensitivity of 89% and a specificity of 83% and a positive predictive value of 81% and negative predictive value of 96% in identifying true cow's milk allergy (37).

For egg allergy, BAT has a sensitivity of 63% and a specificity of 96% for CD203c expression and a sensitivity of 77% and a specificity of 100% for CD63 expression. These numbers are much better compared to performance of skin testing or sIgE for egg (38).

Although these studies show that BAT has potential clinical utility, not all used OFC as the comparator and results are very variable due to variations used in allergen preparations. The use of allergen components may lead to better performance of BAT for these allergens and their clinical correlation.

In tests where basophils do not respond to anti-IgE stimulation, negative results to allergens should generally be considered un-interpretable (39). If basophils are not reactive to the anti-IgE control but show response to the allergen, BAT can be considered positive as long as there is no non-specific activation in the KLH control or other non-allergic control individuals (40).

### 2.2. BAT as a laboratory developed test (LDT) in the United States

The current system of state-regulated testing is known as laboratory developed testing (LDT). In the United States FDA defines an LDT as “(a) laboratory developed test (LDT) is a type of *in vitro* diagnostic test that is designed, manufactured and used within a single laboratory (41). LDTs can be used to measure or detect a wide variety of analytes (substances such as proteins, chemical compounds like glucose or cholesterol, or DNA), in a sample taken from a human body. Some LDTs are relatively simple tests that measure single analytes, such as a test that measures the level of sodium. Other LDTs are complex and may measure or detect one or more analytes” (42). BAT has been developed and used in clinical care as a LDT in the United States.^1^

**Figure 4:**
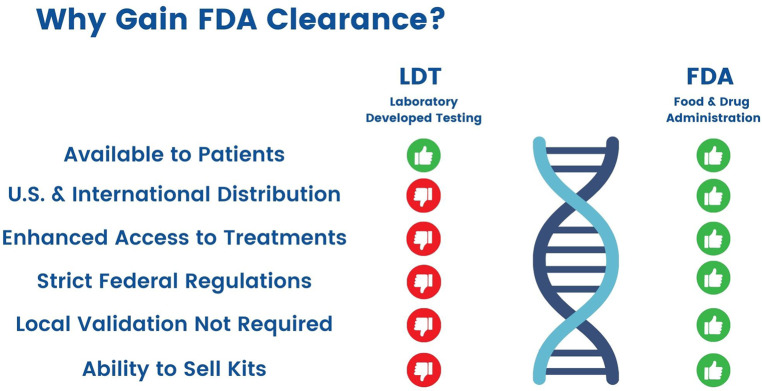
Differences between Laboratory Developed Tests and FDA-Cleared Tests.

The FDA further indicates that “while the uses of an LDT are often the same as the uses of FDA-cleared or approved *in vitro* diagnostic (IVD) tests, the FDA does not consider diagnostic devices to be LDTs if they are designed or manufactured completely, or partly, outside of the laboratory that offers and uses them”. This also implies that LDTs made in an individual laboratory are not sold. Inter-state commerce is an important variable in determining the level of regulation (Figure 4).^1^

The FDA in principle has the authority to intervene in cases in which patient safety is jeopardized. The FDA in most cases has not enforced its authority (enforcement discretion) on LDTs because LDTs have generally been simple laboratory assays or have been used in a very controlled fashion. Laboratory testing personnel and clinicians have also often operated within the same institution or clinical practice (43). This had provided a safety net in clinical utility and potential adverse events related to the testing outcomes.

However, advances in technology and new business models, the FDA has noted, has resulted in more complicated LDTs that present greater risks, and which are more similar to other FDA approved tests that have undergone premarket review. In 2010, the FDA announced its intention to reconsider its enforcement discretion for LDTs. More recently, a bill called the VALID Act has been proposed to increase FDA oversight on LDTs.

There are three components to LDTs in clinical care (44) (Table 1). The first, which is the pre-analytical part, is determined and initiated by the ordering physician. The final post-analytical part is prepared by the clinical pathologist who interprets the results in the context of clinical condition. The analytical part of the test is performed by the laboratory personnel. The analytical steps of laboratory testing are a complex process that starts with the draw to the finalizing of the results for interpretation. Since LDTs, by definition, are developed and used in a single laboratory, regulatory requirements and inspections by the state focuses solely on the analytical part of the testing process (20). In contrast, FDA approved tests can be distributed widely and sold across state lines, and for this purpose need very strictly defined clinical indication and reporting—two important points that will need to be clearly defined for BAT in the process of FDA clearance of an LDT.

**Table 1:** Total testing process.

• **Pre-Analytical**: Test selection ○ Accurate test selection is based on the questions formulated by the ordering physician. ○ Asking the right question for the right issue ○ Interference with various immune targeting medications should be considered ○ This is the part of the test selection that is most prone to errors ○ Right laboratory for the right test should be selected • **Analytical:** ○ Only part of the test that takes place in laboratory proper ○ Only part of the testing that’s covered by certification (e.g., CLIA) and accreditation (e.g., CAP) process. • **Post-Analytical** ○ Interpretation of the assay data ○ Interpretation of the assay results ○ Action plan by the ordering physician ○ Similar to the pre-analytical phase, part of the total testing process most prone to errors

The LDT state-level process has reduced cost as well as the speed such tests are entered into clinical practice. It has also enabled innovation by the rapid identification of new biomarkers facilitating the development of novel therapies. One example is the speedy identification of the role of caspases in COVID-19 pathogenesis, development of this as a laboratory developed test, verification of the findings in clinical samples which led to the completion of a Phase 1 study in COVID in 2021 using a pan-caspase inhibitor (45). Innovations in diagnostics have helped advance many therapies.

## 3. History of flow cytometry-based diagnostics and the FDA

BAT is a flow cytometry-based diagnostic test. The FDA has only approved one such test. Its record of reviewing such tests offers lessons for what is needed for BAT approval.

A major event in the 510(k) regulatory history of clinical flow cytometry occurred in 1997, when the FDA issued the Analyte Specific Reagent (ASR) Rule to provide “assurance that reagents distributed to clinical laboratories by manufacturers for use in clinical assays (in this case LDTs) developed by the laboratories were made under current Good Manufacturing Practices (cGMP)”. Manufacturers of these reagents were required to register with the FDA and list such reagents. FDA also required the reporting of malfunctions, injuries and deaths related to these reagents.^2^

After the publication of the ASR rule in 1997, some manufacturers started bundling individual ASRs together to form reagent cocktails. This conflicted with the definition of the single reagent ASRs rule that the FDA had defined. In 2007, the FDA clarified the intentions of the ASR rule in the Guidance for Industry and FDA Staff on Commercially Distributed Analyte Specific Reagents (ASRs). In the 2007 guidance, the FDA states that “bundling of ASRs into a panel of multi-analytes is inconsistent with the definition of an ASR”. After this guidance, most multi-analyte reagents/cocktails were withdrawn from the market to comply with this new ASR ruling.^2^

The next events in the regulation of clinical flow cytometry were three CDER FDA sponsored public workshops in 2013, on minimal residual disease (MRD) in leukemias and Clinical Flow Cytometry and Hematologic Malignancy. Ultimately, this same approach was applied to the standardization of MRD in plasma cell neoplasms (MM) and resulted in a Special Issue of Clinical Cytometry (46).

A Flow Cytometric Devices Guidance Document was released *via* the Federal Register on October 14, 2015. After several unfavorable comments to the docket, it was withdrawn on February 21, 2015. The major criticism was that it did not address the issues of hematologic malignancies and that it was outdated. Prior to the publication and withdrawal of this second FDA flow cytometry guidance document, there was a consensus document prepared and published by two professional organizations: the International Council for Standardization in Hematology (ICSH) and the International Clinical Cytometry Society (ICCS). These Practice Guidelines (2013) consisted of the following: preanalytical issues; analytical issues; post analytic considerations and assay performance criteria. These Practice Guidelines were submitted to the FDA for review as a recognized standard. The decision was out on hold due to an announcement that Congress was going to pass the Valid Act. A decision concerning these guidelines is still pending.

On June 29, 2017 the FDA approved Beckman Coulter's ClearLLab Reagents, making this the first flow cytometry test that detects leukemias and lymphomas. These reagents were approved so they could be used to screen malignant cells in peripheral whole blood, bone marrow, and lymph node samples. The test has the capability to distinguish among chronic leukemia, non-Hodgkin lymphoma, and myeloma. In an official statement the FDA said that this was “a major step forward for the hematology-oncology community.” That assessment was provided by Alberto Gutierrez, Ph.D., Director of the Office of *In Vitro* Diagnostics and Radiological Health in the FDA's Center for Devices and Radiological Health. “Laboratories and health care professionals now have access to an FDA-validated test that provides consistent results to aid in the diagnoses of these serious cancers,” it added.^3^

FDA evaluated the ClearLLab Reagents through their *de novo* premarket pathway and cleared the test and the reagents based on the tests performance in a clinical trial. The clinical study was performed on 279 patient samples using other detection methods of malignancy as a comparison. The results of this study showed that the assay correctly identified a cancer presence 84.2% of the time which agreed with the clinical trial site's diagnosis in over ninety percent of the cases.

Finally, a proposed down classification for clinical flow cytometers was posted in the Federal Register on March 6, 2019. However, it was put on hold with the onset of the COVID-19 pandemic.^4^

A new Clinical and Laboratory Standards Institute (CLSI) document entitled H62 Validation of Assays Performed by Flow Cytometry was released on October 27, 2021 (47). This document has been submitted to the FDA for consideration as a recognized standard in clinical flow cytometry.

Taking the BAT test through the FDA pathway will ensure reproducibility across laboratories by standardizing the test reagents (antibodies, allergens, etc.). The FDA approval process will ensure standardization of basophil identification in peripheral blood samples, analysis of the data, and a specific indication for the use of BAT. The validation will happen through a multicenter clinical trial.

## 4. VALID act

Since the approval of FDA's Medical Device Amendments in 1976, the agency has tightened its stance on enforcing LDTs, starting to target how certain laboratory tests are used and marketed. The FDA has used its discretion in certain scenarios where it felt the safety and the accuracy of the tests were impacted but not take broad steps to regulate LDTs in general as of now.

There was a significant change in FDA's perspective in 2014 when a draft guidance was published that described the plan to phase out FDA enforcement discretion and to fully regulate LDTs. This guidance led to debate raising concerns about the FDA regulating LDTs, as that is currently performed at the state level. There was also concern that changing a longstanding regulatory policy might result in decrease in innovation and patient care. Lastly, some have questioned whether FDA has the necessary infrastructure to regulate the complex LDT market. Considering these issues, this draft guidance was withdrawn in 2015.

Despite this back and forth, bipartisan support for the VALID has continued to grow, particularly with regard to developing a new statutory authority that would address concerns raised by various stakeholders on FDA's approach. After various legislations failed to advance through Congress, the Senate recently attached the “Verifying Accurate Leading-edge IVCT Development Act of 2022” (the “VALID Act”) to the first draft of a “must pass” user fee legislation. Once the user fee reauthorization draft was introduced in May 2022, the United States Senate prepared a new bill that would re-write FDA regulation of clinical testing. VALID was introduced to the United States Senate by Senators Patty Murray (D-WA) and Richard Burr (R-NC) as a part of the bipartisan FDA Safety and Landmark Advancements Act. Once enacted into law, the plan was for VALID to take effect in October 2027. This would provide the FDA time to transition into the new clinical diagnostics regulation environment.^5^

In a summary statement “The Senate Committee on Health, Education, Labor, and Pensions (HELP) on June 14, 2022 approved a package of bills to reauthorize existing Food & Drug Administration (FDA) user fees and included new legislation (the VALID Act) which would authorize the FDA to regulate *in vitro* diagnostics (IVDs) including laboratory developed tests (LDTs)”. A summary of the key components of the VALID Act is shown in Table 2.^6^

**Table 2:** Key components of the VALID Act.

• Exempting all laboratory-developed tests currently in use through the VALID Act’s “grandfather” exemption. • Laboratories may still introduce LDTs without undergoing premarket review between the VALID Act’s passage and October 1, 2027. • The VALID Act would not be implemented for 5 years with an effective date of October 1, 2027, allowing time to further refine the regulatory framework. • Requires the FDA to conduct public hearings 1 year from date of enactment and publish formal regulations which are subject to public comment within 2 years of enactment. • Directing the FDA to avoid issuing or enforcing regulations or guidance that are duplicative of CLIA. • Offering several exemptions from FDA pre-market review, including those LDTs that are low-risk, low volume, modified tests, manual tests, and humanitarian tests. • Authorizing the FDA to collect user fees and establish a process by which the FDA must negotiate with the laboratory industry to set user fees, including future approval by Congress. • Establishes mitigating measures, such as labeling, performance testing, and clinical studies, to shift higher-risk LDTs to lower tiers of regulation. • It would create a risk-based system of oversight utilizing tiers (low-, moderate-, and high-risk) to target FDA oversight. • It would utilize mitigating measures to shift LDTs into lower tiers of regulation. These measures would include such practices as appropriate labeling, performance testing, submission of clinical data, clinical studies, and posting information on a website. • It would prohibit the FDA from infringing on the practice of medicine.

At the time of writing of this manuscript, deliberations in both the Senate and the House resulted in a decision to delay the authorization of the law to a later date.

Even though the VALID act may not pass during the current United States administration, it is clear that the discussion will continue. Engaging with the FDA to approve tests such as BAT, will allow for a better understanding of the process of the clearance of the use of flow cytometry for different indications as the regulatory landscape for laboratory testing goes through changes in the United States.

### 4.1. Where do we go from here? A path to FDA cleared BAT

The best guidance that is currently available for developing FDA cleared flow cytometry based testing comes from the September ICCS 2020 virtual meeting. There were two presentations, which addressed minimal residual disease (MRD) detection.

The first was by Doug Jeffery, PhD of IVDx Consulting, LLC, titled “Flow cytometry-based minimal residual disease analysis assays submitted for FDA Clearance: Regulatory Perspective”. The presentation outlined three regulatory pathways for clinical flow cytometry assays: (1) LDTs, (2) Investigational Device [Exemption (IDE)] and (3) the IVD 510(k)/*de novo*. LDTs were and remain under enforcement discretion. Of these, the IVD pathway is more demanding in that it must be determined to be substantially equivalent to a predicate device. If there is no predicate device, then the *de novo* pathway is necessary.

The second presentation by Horatiu Olteanu, MD, PhD, Professor and Medical Director, Cell Kinetics Laboratory, Mayo Clinic, Rochester, MN was titled “Flow Cytometry-Based Minimal Residual Disease Analysis Assays Submitted for FDA Clearance: A Laboratory Perspective”. It was a personal assessment from his perspective as medical director of the flow cytometry laboratory, based on two MRD flow assays submitted for FDA IDE clearance as part of two different clinical trials. The same flow cytometric assay, the consensus EURO Flow two tube 8-color assay was used in both studies. One clinical study involved a treatment decision in treated MM with or without MRD. The second clinical trial involved patients with high risk CLL, and continued treatment was determined by the presence of MRD. The FDA determined that there was significant risk in the MM study thereby necessitating the submission of an IDE. In the CLL study, the FDA determined that there was a non-significant risk. The FDA did note that they thought the recently published European Research Initiative Consortium (ERIC) single tube, 10 color MRD assay was superior to the two tube Euro Flow panel and less costly. FDA recommended that in a future submission, the sponsor should consider using the ERIC panel over the EuroFlow panel. If the future submission were to contain a therapeutic indication, banked specimens were recommended (55–57). The recommendations of these two speakers point to a do list for the clearance of BAT through the FDA (summarized in Table 3).

**Table 3:** Regulatory clearance pathway for a BAT kit.

• **Generating summary of LDT data on peanut BAT performed in laboratories across the United States** • **Discussions with the FDA:** ○ Regulatory pathway; *de novo*, 520 (k), analytical, exemption, registration ○ Assay specific endpoints ○ Clinical trial design ○ Sample size determination ○ Flow cytometry device down-regulation • **Standardization of Allergen Extracts** • **Analyte Specific Reagent generation** • **Transportation logistics of blood samples** • **Securing intellectual property on preparation of BAT samples and assay preparation** • **Securing funding** ○ Crowd-funding; medical and non-medical community ○ Other conventional sources; angel, venture capital, pharma

#### 4.1.1. Summary of LDT data

For a CLIA or CAP certified laboratory currently performing BAT as an LDT, including a summary of the results of the LDTs for potential FDA review will be critical. The data includes basophil identification (manuscript in preparation), activation, as well as the performance of the testing with clinical correlation. Such a summary along with an SOP should already be in place for both CLIA and or CAP inspection of the LDTs. This will provide the FDA information on the performance of the test and help in the design of the study for FDA clearance.

#### 4.1.2. Discussions with the FDA

Any FDA clearance path will also require a clinical trial to support the indication for the test. Such a trial should be designed only after discussions with the FDA on the technical aspects, indication for the use of the test as well as patient size of the clinical study.

#### 4.1.3. Generation of a standardized allergen extract

Good Manufacturing Practice (GMP) to standardize allergens for use in diagnosis and treatment is a critical regulatory requirement by the FDA. Allergens are derived from natural sources. Their manufacturing may involve roasting, grinding, defatting, extraction, clarification, and sterilization that results in allergen heterogeneity. A consistency within the manufacturing process will improve the efficacy and the safety of the BAT. Sourcing of peanut flour for BAT from a food-grade peanut manufacturer will be the starting point for test substance manufacturing.

The clinical correlation of recombinant molecules (i.e., Ara h 1, Ara h 2, etc.) versus native peanut allergen has not been studied as extensively, but may provide added value in clinical correlations.

#### 4.1.4. Generation of analyte specific reagents to be used in BAT

ASRs are raw materials and components that are used to develop a laboratory assay. By definition, the key characteristic of each component of an ASR is its ability to attach to or react with a substance whose detection is clinically meaningful.

ASR rule requires that manufacturers list proprietary name, common name, and quantity or concentration of the reagent; the source and a measure of its activity; and the name and place of business of the manufacturer. There also needs to be an establishment of registration, device listing, and compliance with FDA's quality system regulation, medical device reporting requirements, and ASR labeling and distribution requirements.

The ASRs for BAT will include antibodies, antigens (e.g., peanut and control allergen) as well as reagents that stimulate basophils (e.g., fMLP, anti-IgE).

#### 4.1.5. Transporting the blood sample

This can be a potential hurdle for clinical BAT. Temperature control boxes, choice of anticoagulant (heparin is the preferred option), time frame before the test would become invalid will need to be part of the clinical trial readout. In a recent paper, we demonstrated very minimal impact of transport on blood samples (33).

#### 4.1.6. Securing intellectual property

For patents related to diagnostic subject matter, U.S. case law stipulates there are several types of claims to try to meet eligibility. These include (a) method of preparing samples for analysis, (b) method of diagnosing + treating, (c) A set of assay samples, (d) a kit and (e) a method of diagnosing for ex-US filings are important to consider.

#### 4.1.7. Securing funding

Recent developments in fundraising options (e.g., crowdfunding) allowing the greater allergy and patient community to invest will facilitate such a testing process to go through the clinical trials and regulatory process, in the absence of pharmaceutical, government or device manufacturer backing. With the Jumpstart Our Business Startups (JOBS) Act of 2012, signed into law by President Barack Obama on April 5 of that year, equity crowdfunding has emerged as a viable source for early-stage seed capital. Under Title III of the JOBS Act of 2012, early stage ventures could raise a maximum of $1.07 million in a 12-month period from both accredited and non-accredited investors, so long as the funding round in question is hosted on a Financial Industry Regulatory Authority (FINRA)-approved crowdfunding portal. On March 15, 2021, this maximum was increased to $5 million per 12 month period. A total of $486.8 million was raised in 2021through 1,448 individual regulation crowdfunding (also referred to as RegCF) rounds. The crowdfunding investment market has held firm despite recent economic weakness, with $235.1 million investing *via* regulation crowdfunding (RegCF) in the first half of 2022, compared with $219.4 million in the same period in 2021.

## 5. Conclusion

BAT has been used as a research test now for over 30 years. Over the past 4 years, this test has been validated for use in diagnosing and monitoring food allergies as a laboratory developed test in the United States. Given the increase in demand for BAT from clinics treating patients with food allergies as well as many centers looking to develop their own LDTs for this test, it is time for its standardization and FDA clearance. FDA acceptance of the first peanut OIT in 2017 and increasing use of BAT in clinical trials of emerging food allergy therapeutics are additional reasons for pursuing agency approval for this test. Establishing BAT as a platform to test many food allergens and the standardization of the reagents and food antigens used in this assay will improve patient care as well as research in food allergies.

## Data Availability

The raw data supporting the conclusions of this article will be made available by the authors, without undue reservation.

## Footnotes

^1^ https://www.fda.gov/medical-devices/in-vitro-diagnostics/laboratory-developed-tests

^2^ https://www.fda.gov/regulatory-information/search-fda-guidance-documents/commercially-distributed-analyte-specific-reagents-asrs-frequently-asked-questions

^3^ https://www.fda.gov/news-events/press-announcements/fda-allows-marketing-test-aid-detection-certain-leukemias-and-lymphomas

^4^ https://www.federalregister.gov/documents/2019/03/06/2019-03967/medical-devices-exemption-from-premarket-notification-class-ii-devices-flow-cytometer-instruments

^5^ https://www.cap.org/advocacy/latest-news-and-practice-data/june-15-2022

^6^ https://www.help.senate.gov/hearings/s-4348-s-958-s-4353-hr-1193-and-s-4053
